# *N*,*N*-Dicyclo­hexyl-2-(5,7-dichloro-8-quinol­yloxy)acetamide

**DOI:** 10.1107/S160053680903966X

**Published:** 2009-10-07

**Authors:** Jing-Lin Wang

**Affiliations:** a Department of Biology and Chemistry, Changzhi University, Changzhi, Shanxi 046011, People’s Republic of China

## Abstract

The mol­ecular and crystal structures of the title compound, C_23_H_28_Cl_2_N_2_O_2_, are very close to those of the bromine-substituted analogue *N*,*N*-dicyclo­hexyl-2-(5,7-dibromo-8-quinol­yloxy)acetamide. The two cyclo­hexyl groups adopt normal chair conformation. The amide N and C atoms have a planar configuration. The crystal packing is stabilized by inter­molecular C—H⋯O hydrogen bonds and aromatic π⋯π stacking inter­actions [centroid–centroid separation = 3.5715 (4) Å for symmetry-related pyridine rings]. In addition, the crystal structure exhibits Cl⋯Cl halogen contacts of 3.4675 (3) Å.

## Related literature

For background to the applications of 8-hydroxy­quinoline and its derivatives, see: Bratzel *et al.* (1972 ▶); Hanna *et al.* (2002 ▶); Pierre *et al.* (2003 ▶); Tang *et al.* (1987 ▶); Zeng *et al.* (2006 ▶). For structures of 8-hydroxy­quinolinate amide compounds, see: Bi *et al.* (2007 ▶); Tang *et al.* (2007 ▶); Liu *et al.* (2007 ▶).
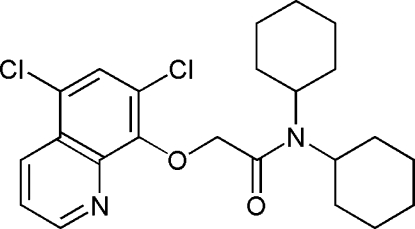


## Experimental

### 

#### Crystal data

C_23_H_28_Cl_2_N_2_O_2_
*M* *_r_* = 435.37
Triclinic, 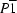

*a* = 9.8476 (11) Å
*b* = 10.7542 (12) Å
*c* = 11.1376 (12) Å
α = 72.392 (2)°
β = 86.880 (2)°
γ = 80.208 (2)°
*V* = 1107.9 (2) Å^3^
*Z* = 2
Mo *K*α radiation
μ = 0.32 mm^−1^
*T* = 293 K
0.22 × 0.20 × 0.18 mm

#### Data collection

Bruker APEXII CCD area-detector diffractometer
Absorption correction: multi-scan (*SADABS*; Sheldrick,1996 ▶) *T*_min_ = 0.934, *T*_max_ = 0.946
5942 measured reflections
4088 independent reflections
3400 reflections with *I* > 2σ(*I*)
*R*_int_ = 0.018

#### Refinement

*R*[*F* ^2^ > 2σ(*F* ^2^)] = 0.042
*wR*(*F* ^2^) = 0.112
*S* = 1.02
4088 reflections
262 parameters
H-atom parameters constrained
Δρ_max_ = 0.33 e Å^−3^
Δρ_min_ = −0.24 e Å^−3^

### 

Data collection: *APEX2* (Bruker, 2001 ▶); cell refinement: *SAINT* (Bruker, 2001 ▶); data reduction: *SAINT*; program(s) used to solve structure: *SHELXTL* (Sheldrick, 2008 ▶); program(s) used to refine structure: *SHELXTL*; molecular graphics: *SHELXTL*; software used to prepare material for publication: *SHELXTL*.

## Supplementary Material

Crystal structure: contains datablocks I, global. DOI: 10.1107/S160053680903966X/bh2246sup1.cif

Structure factors: contains datablocks I. DOI: 10.1107/S160053680903966X/bh2246Isup2.hkl

Additional supplementary materials:  crystallographic information; 3D view; checkCIF report

## Figures and Tables

**Table 1:** Hydrogen-bond geometry (Å, °)

*D*—H⋯*A*	*D*—H	H⋯*A*	*D*⋯*A*	*D*—H⋯*A*
C6—H6*A*⋯O2^i^	0.93	2.50	3.414 (3)	169
C10—H10*B*⋯O2^ii^	0.97	2.38	3.323 (2)	164

Symmetry codes: (i) 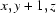; (ii) 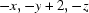.

**Fig. 1.**
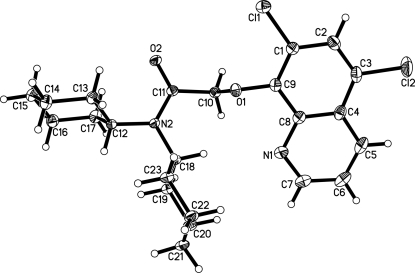
The molecular structure of (I), with atom labels and 30% probability displacement ellipsoids.

**Fig. 2.**
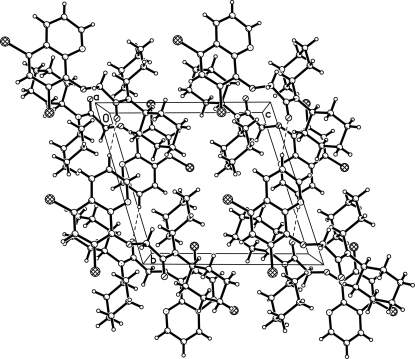
The packing diagram of (I), viewed down the a axis, showing the intermolecular hydrogen bonds (dashed lines).

## supplementary crystallographic information

### Comment

8-Hydroxyquinoline and its derivatives have been used widely in analytical chemistry (Bratzel *et al.*, 1972), coordination chemistry (Hanna *et al.*, 2002), pharmaceutical chemistry (Pierre *et al.*, 2003), materials chemistry (Tang *et al.*, 1987) and many other topics. The synthesis and the development of novel 8-hydroxyquinoline derivatives have been a significant research subject (Zeng *et al.*, 2006). Recently, the structures of 8-hydroxyquinolinate amide-type compounds, namely, *N*,*N*-diphenyl-2-(5,7-dibromoquinolin-8-yloxy)acetamide (Bi *et al.*, 2007), *N*,*N*-diphenyl-2-(5,7-dichloroquinolin-8-yloxy)acetamide (Tang *et al.*, 2007), and *N*,*N*-dicyclohexyl-2-(5,7-dibromoquinolin-8-yloxy)acetamide (Liu *et al.*, 2007) have been reported. Here, we report the synthesis and crystal structure of the title compound, (I, Fig. 1), a new amide-based 5,7-dichloro-8-hydroxyquinoline derivative.

All bond lengths and angles in (I) are within normal ranges and comparable with those in the related above-cited compounds. Compound (I) has the same crystal form as the bromine analogue. The quinoline fragment is essentially planar, with a dihedral angle of 0.35 (9)° between the benzene (C1···C4/C8/C9) ring and pyridine (N1/C4···C8) ring. The two cyclohexyl groups adopt the normal chair conformation. The amide N and C atoms have a planar configuration. The crystal packing exhibits intermolecular C6—H6···O2 and C10—H10···O2 hydrogen bonds (Table 1 and Fig. 2), and π···π interactions [shortest centroid-centroid separation = 3.5715 (4) Å] between the pyridine rings of the neighbouring molecules. In addition, the crystal structure exhibits Cl···Cl halogen contacts of 3.4675 (3) Å.

### Experimental

To a solution of 5,7-dichloro-8-hydroxyquinoline (4.18 g, 20 mmol) in acetone (60 ml) were added 2-chloro-*N*,*N*-dicyclohexylacetamide (5.16 g, 20 mmol), K_2_CO_3_ (3.04 g, 22 mmol) and KI (0.5 g), and the resulting mixture was refluxed for 5 h. After cooling to room temperature, the mixture was washed three times with water and filtered. The filter cake was collected and purified by recrystallization with a mixture of ethanol/water. Colourless single crystals of (I) suitable for X-ray diffraction study were obtained by slow evaporation of an ethanol solution over a period of 15 d.

### Refinement

All H atoms were located in a difference Fourier map and constrained to ride on their parent atoms, with C—H = 0.95–0.99 Å and with *U*_iso_(H) = 1.2 *U*_eq_(C).

### Crystal data

**Table:**

C_23_H_28_Cl_2_N_2_O_2_	*Z* = 2
*M**_r_* = 435.37	*F*(000) = 460
Triclinic, *P*1	*D*_x_ = 1.305 Mg m^−^^3^
Hall symbol: -P 1	Mo *K*α radiation, λ = 0.71073 Å
*a* = 9.8476 (11) Å	Cell parameters from 2570 reflections
*b* = 10.7542 (12) Å	θ = 2.7–25.7°
*c* = 11.1376 (12) Å	µ = 0.32 mm^−^^1^
α = 72.392 (2)°	*T* = 293 K
β = 86.880 (2)°	Rhombus, colourless
γ = 80.208 (2)°	0.22 × 0.20 × 0.18 mm
*V* = 1107.9 (2) Å^3^	

### Data collection

**Table:**

Bruker APEXII CCD area-detector diffractometer	4088 independent reflections
Radiation source: fine-focus sealed tube	3400 reflections with *I* > 2σ(*I*)
graphite	*R*_int_ = 0.018
φ and ω scans	θ_max_ = 25.7°, θ_min_ = 1.9°
Absorption correction: multi-scan (*SADABS*; Sheldrick,1996)	*h* = −11→11
*T*_min_ = 0.934, *T*_max_ = 0.946	*k* = −13→10
5942 measured reflections	*l* = −13→11

### Refinement

**Table:**

Refinement on *F*^2^	Primary atom site location: structure-invariant direct methods
Least-squares matrix: full	Secondary atom site location: difference Fourier map
*R*[*F*^2^ > 2σ(*F*^2^)] = 0.042	Hydrogen site location: inferred from neighbouring sites
*wR*(*F*^2^) = 0.112	H-atom parameters constrained
*S* = 1.02	*w* = 1/[σ^2^(*F*_o_^2^) + (0.0584*P*)^2^ + 0.3083*P*] where *P* = (*F*_o_^2^ + 2*F*_c_^2^)/3
4088 reflections	(Δ/σ)_max_ = 0.001
262 parameters	Δρ_max_ = 0.33 e Å^−^^3^
0 restraints	Δρ_min_ = −0.24 e Å^−^^3^
0 constraints	

### Fractional atomic coordinates and isotropic or equivalent isotropic displacement parameters (Å^2^)

**Table:**

	*x*	*y*	*z*	*U*_iso_*/*U*_eq_	
Cl1	0.10108 (5)	0.94426 (5)	0.29221 (5)	0.03764 (15)	
Cl2	−0.02741 (8)	1.39471 (7)	0.41588 (7)	0.0698 (2)	
O1	0.22307 (12)	1.10728 (12)	0.06239 (11)	0.0293 (3)	
O2	0.16900 (12)	0.91089 (12)	−0.08811 (12)	0.0314 (3)	
N1	0.21941 (17)	1.37466 (16)	−0.00224 (16)	0.0359 (4)	
N2	0.32140 (14)	1.04756 (14)	−0.18351 (13)	0.0243 (3)	
C1	0.10394 (18)	1.11220 (19)	0.25299 (17)	0.0299 (4)	
C2	0.04676 (19)	1.1801 (2)	0.33885 (19)	0.0373 (5)	
H2A	0.0100	1.1348	0.4150	0.045*	
C3	0.0459 (2)	1.3124 (2)	0.3093 (2)	0.0400 (5)	
C4	0.10273 (19)	1.3853 (2)	0.19466 (19)	0.0349 (5)	
C5	0.1058 (2)	1.5229 (2)	0.1581 (2)	0.0444 (5)	
H5A	0.0684	1.5730	0.2105	0.053*	
C6	0.1636 (2)	1.5813 (2)	0.0463 (2)	0.0473 (6)	
H6A	0.1666	1.6715	0.0210	0.057*	
C7	0.2187 (2)	1.5027 (2)	−0.0303 (2)	0.0439 (5)	
H7A	0.2577	1.5442	−0.1067	0.053*	
C8	0.16118 (18)	1.31440 (19)	0.11007 (18)	0.0301 (4)	
C9	0.16069 (17)	1.17641 (18)	0.14113 (17)	0.0277 (4)	
C10	0.13960 (17)	1.11664 (18)	−0.04396 (17)	0.0263 (4)	
H10A	0.1299	1.2052	−0.1025	0.032*	
H10B	0.0485	1.0971	−0.0158	0.032*	
C11	0.21283 (17)	1.01619 (17)	−0.10754 (16)	0.0249 (4)	
C12	0.38451 (17)	0.96001 (17)	−0.25850 (16)	0.0246 (4)	
H12A	0.4570	1.0035	−0.3095	0.030*	
C13	0.45558 (18)	0.82518 (18)	−0.17828 (17)	0.0284 (4)	
H13A	0.3882	0.7785	−0.1242	0.034*	
H13B	0.5241	0.8370	−0.1251	0.034*	
C14	0.5251 (2)	0.7433 (2)	−0.26245 (19)	0.0364 (5)	
H14A	0.5999	0.7849	−0.3089	0.044*	
H14B	0.5638	0.6558	−0.2102	0.044*	
C15	0.4236 (2)	0.73116 (19)	−0.35466 (19)	0.0360 (5)	
H15A	0.3540	0.6814	−0.3084	0.043*	
H15B	0.4718	0.6829	−0.4093	0.043*	
C16	0.3540 (2)	0.8660 (2)	−0.43435 (18)	0.0351 (4)	
H16A	0.2867	0.8548	−0.4891	0.042*	
H16B	0.4223	0.9126	−0.4869	0.042*	
C17	0.28254 (19)	0.94781 (19)	−0.35147 (17)	0.0322 (4)	
H17A	0.2076	0.9060	−0.3054	0.039*	
H17B	0.2439	1.0352	−0.4040	0.039*	
C18	0.37854 (17)	1.17059 (17)	−0.19956 (16)	0.0246 (4)	
H18A	0.3305	1.2136	−0.1397	0.029*	
C19	0.35073 (19)	1.26735 (18)	−0.33206 (17)	0.0303 (4)	
H19A	0.3936	1.2264	−0.3942	0.036*	
H19B	0.2523	1.2884	−0.3475	0.036*	
C20	0.4082 (2)	1.39395 (19)	−0.34509 (19)	0.0364 (5)	
H20A	0.3579	1.4397	−0.2893	0.044*	
H20B	0.3948	1.4516	−0.4308	0.044*	
C21	0.5614 (2)	1.36517 (19)	−0.31312 (19)	0.0364 (5)	
H21A	0.6130	1.3274	−0.3739	0.044*	
H21B	0.5934	1.4470	−0.3181	0.044*	
C22	0.5870 (2)	1.26914 (19)	−0.18086 (18)	0.0336 (4)	
H22A	0.6851	1.2489	−0.1638	0.040*	
H22B	0.5422	1.3102	−0.1195	0.040*	
C23	0.53174 (18)	1.14179 (18)	−0.16782 (17)	0.0290 (4)	
H23A	0.5456	1.0840	−0.0822	0.035*	
H23B	0.5822	1.0967	−0.2240	0.035*	

### Atomic displacement parameters (Å^2^)

**Table:**

	*U*^11^	*U*^22^	*U*^33^	*U*^12^	*U*^13^	*U*^23^
Cl1	0.0375 (3)	0.0326 (3)	0.0393 (3)	−0.0063 (2)	−0.0005 (2)	−0.0051 (2)
Cl2	0.0808 (5)	0.0768 (5)	0.0705 (4)	−0.0121 (4)	0.0222 (4)	−0.0538 (4)
O1	0.0266 (6)	0.0312 (7)	0.0302 (7)	0.0007 (5)	−0.0003 (5)	−0.0123 (5)
O2	0.0284 (7)	0.0265 (7)	0.0431 (8)	−0.0122 (5)	0.0090 (6)	−0.0135 (6)
N1	0.0348 (9)	0.0320 (9)	0.0406 (9)	−0.0091 (7)	−0.0001 (7)	−0.0084 (7)
N2	0.0248 (7)	0.0204 (8)	0.0297 (8)	−0.0077 (6)	0.0051 (6)	−0.0090 (6)
C1	0.0253 (9)	0.0323 (10)	0.0330 (10)	−0.0056 (7)	−0.0024 (7)	−0.0103 (8)
C2	0.0320 (10)	0.0505 (13)	0.0334 (10)	−0.0103 (9)	0.0040 (8)	−0.0170 (9)
C3	0.0349 (10)	0.0507 (14)	0.0439 (12)	−0.0047 (9)	0.0025 (9)	−0.0299 (10)
C4	0.0282 (10)	0.0358 (11)	0.0457 (12)	−0.0021 (8)	−0.0051 (8)	−0.0205 (9)
C5	0.0383 (11)	0.0362 (12)	0.0661 (15)	−0.0001 (9)	−0.0099 (10)	−0.0275 (11)
C6	0.0449 (12)	0.0289 (11)	0.0694 (16)	−0.0073 (9)	−0.0133 (11)	−0.0136 (11)
C7	0.0446 (12)	0.0353 (12)	0.0507 (13)	−0.0144 (9)	−0.0026 (10)	−0.0065 (10)
C8	0.0226 (9)	0.0312 (10)	0.0376 (10)	−0.0035 (7)	−0.0044 (8)	−0.0115 (8)
C9	0.0209 (8)	0.0324 (10)	0.0314 (9)	−0.0018 (7)	−0.0020 (7)	−0.0128 (8)
C10	0.0218 (8)	0.0265 (10)	0.0315 (9)	−0.0054 (7)	0.0019 (7)	−0.0094 (7)
C11	0.0213 (8)	0.0244 (10)	0.0287 (9)	−0.0050 (7)	−0.0012 (7)	−0.0066 (7)
C12	0.0242 (9)	0.0239 (9)	0.0290 (9)	−0.0092 (7)	0.0057 (7)	−0.0108 (7)
C13	0.0286 (9)	0.0278 (10)	0.0301 (9)	−0.0047 (7)	−0.0024 (7)	−0.0103 (8)
C14	0.0357 (10)	0.0316 (11)	0.0418 (11)	0.0012 (8)	−0.0016 (9)	−0.0139 (9)
C15	0.0465 (11)	0.0297 (11)	0.0371 (11)	−0.0106 (9)	0.0046 (9)	−0.0163 (9)
C16	0.0421 (11)	0.0367 (11)	0.0306 (10)	−0.0092 (9)	−0.0026 (8)	−0.0140 (8)
C17	0.0322 (10)	0.0329 (11)	0.0321 (10)	−0.0030 (8)	−0.0050 (8)	−0.0109 (8)
C18	0.0250 (9)	0.0209 (9)	0.0291 (9)	−0.0074 (7)	0.0046 (7)	−0.0082 (7)
C19	0.0298 (9)	0.0258 (10)	0.0340 (10)	−0.0071 (7)	−0.0012 (8)	−0.0053 (8)
C20	0.0455 (12)	0.0231 (10)	0.0362 (11)	−0.0087 (8)	0.0017 (9)	−0.0009 (8)
C21	0.0440 (11)	0.0293 (11)	0.0392 (11)	−0.0203 (9)	0.0064 (9)	−0.0086 (8)
C22	0.0355 (10)	0.0313 (11)	0.0376 (11)	−0.0150 (8)	0.0001 (8)	−0.0104 (8)
C23	0.0294 (9)	0.0252 (10)	0.0315 (9)	−0.0093 (7)	−0.0017 (8)	−0.0045 (8)

### Geometric parameters (Å, °)

**Table:**

Cl1—C1	1.730 (2)	C13—H13A	0.9700
Cl2—C3	1.743 (2)	C13—H13B	0.9700
O1—C9	1.371 (2)	C14—C15	1.519 (3)
O1—C10	1.447 (2)	C14—H14A	0.9700
O2—C11	1.234 (2)	C14—H14B	0.9700
N1—C7	1.315 (3)	C15—C16	1.520 (3)
N1—C8	1.368 (2)	C15—H15A	0.9700
N2—C11	1.351 (2)	C15—H15B	0.9700
N2—C12	1.480 (2)	C16—C17	1.527 (3)
N2—C18	1.483 (2)	C16—H16A	0.9700
C1—C9	1.370 (3)	C16—H16B	0.9700
C1—C2	1.410 (3)	C17—H17A	0.9700
C2—C3	1.358 (3)	C17—H17B	0.9700
C2—H2A	0.9300	C18—C23	1.526 (2)
C3—C4	1.422 (3)	C18—C19	1.534 (2)
C4—C5	1.416 (3)	C18—H18A	0.9800
C4—C8	1.424 (3)	C19—C20	1.526 (3)
C5—C6	1.357 (3)	C19—H19A	0.9700
C5—H5A	0.9300	C19—H19B	0.9700
C6—C7	1.402 (3)	C20—C21	1.527 (3)
C6—H6A	0.9300	C20—H20A	0.9700
C7—H7A	0.9300	C20—H20B	0.9700
C8—C9	1.419 (3)	C21—C22	1.527 (3)
C10—C11	1.527 (2)	C21—H21A	0.9700
C10—H10A	0.9700	C21—H21B	0.9700
C10—H10B	0.9700	C22—C23	1.522 (2)
C12—C13	1.526 (2)	C22—H22A	0.9700
C12—C17	1.529 (2)	C22—H22B	0.9700
C12—H12A	0.9800	C23—H23A	0.9700
C13—C14	1.532 (3)	C23—H23B	0.9700
			
C9—O1—C10	114.18 (13)	C13—C14—H14B	109.3
C7—N1—C8	117.15 (18)	H14A—C14—H14B	108.0
C11—N2—C12	119.90 (14)	C14—C15—C16	111.50 (16)
C11—N2—C18	122.60 (14)	C14—C15—H15A	109.3
C12—N2—C18	117.46 (13)	C16—C15—H15A	109.3
C9—C1—C2	121.40 (18)	C14—C15—H15B	109.3
C9—C1—Cl1	120.29 (15)	C16—C15—H15B	109.3
C2—C1—Cl1	118.31 (15)	H15A—C15—H15B	108.0
C3—C2—C1	119.38 (18)	C15—C16—C17	110.99 (15)
C3—C2—H2A	120.3	C15—C16—H16A	109.4
C1—C2—H2A	120.3	C17—C16—H16A	109.4
C2—C3—C4	122.17 (18)	C15—C16—H16B	109.4
C2—C3—Cl2	118.59 (16)	C17—C16—H16B	109.4
C4—C3—Cl2	119.24 (16)	H16A—C16—H16B	108.0
C5—C4—C3	125.15 (19)	C16—C17—C12	110.61 (15)
C5—C4—C8	117.42 (19)	C16—C17—H17A	109.5
C3—C4—C8	117.43 (18)	C12—C17—H17A	109.5
C6—C5—C4	119.7 (2)	C16—C17—H17B	109.5
C6—C5—H5A	120.2	C12—C17—H17B	109.5
C4—C5—H5A	120.2	H17A—C17—H17B	108.1
C5—C6—C7	118.6 (2)	N2—C18—C23	111.79 (14)
C5—C6—H6A	120.7	N2—C18—C19	111.53 (14)
C7—C6—H6A	120.7	C23—C18—C19	111.35 (14)
N1—C7—C6	125.0 (2)	N2—C18—H18A	107.3
N1—C7—H7A	117.5	C23—C18—H18A	107.3
C6—C7—H7A	117.5	C19—C18—H18A	107.3
N1—C8—C9	117.69 (17)	C20—C19—C18	110.40 (15)
N1—C8—C4	122.22 (18)	C20—C19—H19A	109.6
C9—C8—C4	120.09 (17)	C18—C19—H19A	109.6
C1—C9—O1	120.48 (17)	C20—C19—H19B	109.6
C1—C9—C8	119.52 (17)	C18—C19—H19B	109.6
O1—C9—C8	119.92 (16)	H19A—C19—H19B	108.1
O1—C10—C11	107.15 (13)	C19—C20—C21	111.63 (16)
O1—C10—H10A	110.3	C19—C20—H20A	109.3
C11—C10—H10A	110.3	C21—C20—H20A	109.3
O1—C10—H10B	110.3	C19—C20—H20B	109.3
C11—C10—H10B	110.3	C21—C20—H20B	109.3
H10A—C10—H10B	108.5	H20A—C20—H20B	108.0
O2—C11—N2	123.20 (16)	C22—C21—C20	110.66 (16)
O2—C11—C10	118.40 (15)	C22—C21—H21A	109.5
N2—C11—C10	118.39 (15)	C20—C21—H21A	109.5
N2—C12—C13	113.45 (14)	C22—C21—H21B	109.5
N2—C12—C17	112.06 (14)	C20—C21—H21B	109.5
C13—C12—C17	112.03 (15)	H21A—C21—H21B	108.1
N2—C12—H12A	106.2	C23—C22—C21	110.94 (16)
C13—C12—H12A	106.2	C23—C22—H22A	109.5
C17—C12—H12A	106.2	C21—C22—H22A	109.5
C12—C13—C14	110.40 (15)	C23—C22—H22B	109.5
C12—C13—H13A	109.6	C21—C22—H22B	109.5
C14—C13—H13A	109.6	H22A—C22—H22B	108.0
C12—C13—H13B	109.6	C22—C23—C18	110.84 (15)
C14—C13—H13B	109.6	C22—C23—H23A	109.5
H13A—C13—H13B	108.1	C18—C23—H23A	109.5
C15—C14—C13	111.47 (16)	C22—C23—H23B	109.5
C15—C14—H14A	109.3	C18—C23—H23B	109.5
C13—C14—H14A	109.3	H23A—C23—H23B	108.1
C15—C14—H14B	109.3		
			
C9—C1—C2—C3	1.0 (3)	C12—N2—C11—O2	6.4 (2)
Cl1—C1—C2—C3	−179.00 (15)	C18—N2—C11—O2	−175.96 (16)
C1—C2—C3—C4	−0.9 (3)	C12—N2—C11—C10	−172.74 (14)
C1—C2—C3—Cl2	179.13 (15)	C18—N2—C11—C10	4.9 (2)
C2—C3—C4—C5	−179.75 (19)	O1—C10—C11—O2	102.13 (17)
Cl2—C3—C4—C5	0.2 (3)	O1—C10—C11—N2	−78.66 (18)
C2—C3—C4—C8	0.2 (3)	C11—N2—C12—C13	−66.8 (2)
Cl2—C3—C4—C8	−179.80 (14)	C18—N2—C12—C13	115.52 (16)
C3—C4—C5—C6	179.7 (2)	C11—N2—C12—C17	61.3 (2)
C8—C4—C5—C6	−0.3 (3)	C18—N2—C12—C17	−116.39 (16)
C4—C5—C6—C7	0.1 (3)	N2—C12—C13—C14	−176.85 (14)
C8—N1—C7—C6	0.3 (3)	C17—C12—C13—C14	55.0 (2)
C5—C6—C7—N1	−0.2 (3)	C12—C13—C14—C15	−54.8 (2)
C7—N1—C8—C9	179.69 (17)	C13—C14—C15—C16	56.0 (2)
C7—N1—C8—C4	−0.5 (3)	C14—C15—C16—C17	−56.3 (2)
C5—C4—C8—N1	0.4 (3)	C15—C16—C17—C12	55.7 (2)
C3—C4—C8—N1	−179.53 (17)	N2—C12—C17—C16	175.45 (14)
C5—C4—C8—C9	−179.73 (17)	C13—C12—C17—C16	−55.7 (2)
C3—C4—C8—C9	0.3 (3)	C11—N2—C18—C23	123.66 (17)
C2—C1—C9—O1	176.09 (16)	C12—N2—C18—C23	−58.69 (19)
Cl1—C1—C9—O1	−3.9 (2)	C11—N2—C18—C19	−110.94 (18)
C2—C1—C9—C8	−0.5 (3)	C12—N2—C18—C19	66.71 (19)
Cl1—C1—C9—C8	179.51 (13)	N2—C18—C19—C20	178.92 (14)
C10—O1—C9—C1	101.90 (19)	C23—C18—C19—C20	−55.4 (2)
C10—O1—C9—C8	−81.49 (19)	C18—C19—C20—C21	55.5 (2)
N1—C8—C9—C1	179.70 (16)	C19—C20—C21—C22	−56.2 (2)
C4—C8—C9—C1	−0.1 (3)	C20—C21—C22—C23	56.5 (2)
N1—C8—C9—O1	3.1 (2)	C21—C22—C23—C18	−56.7 (2)
C4—C8—C9—O1	−176.79 (15)	N2—C18—C23—C22	−178.16 (14)
C9—O1—C10—C11	−170.23 (14)	C19—C18—C23—C22	56.3 (2)

### Hydrogen-bond geometry (Å, °)

**Table:**

*D*—H···*A*	*D*—H	H···*A*	*D*···*A*	*D*—H···*A*
C6—H6A···O2^i^	0.93	2.50	3.414 (3)	169
C10—H10B···O2^ii^	0.97	2.38	3.323 (2)	164

Symmetry codes: (i) *x*, *y*+1, *z*; (ii) −*x*, −*y*+2, −*z*.
